# Betaine Improves Milk Yield in Grazing Dairy Cows Supplemented with Concentrates at High Temperatures

**DOI:** 10.3390/ani9020057

**Published:** 2019-02-13

**Authors:** Frank R. Dunshea, Kehinde Oluboyede, Kristy DiGiacomo, Brian J. Leury, Jeremy J. Cottrell

**Affiliations:** Faculty of Veterinary and Agricultural Sciences, The University of Melbourne, Parkville, Victoria 3010, Australia; kehinde.oluboyede@sydney.edu.au (K.O.); kristyd@unimelb.edu.au (K.D.); brianjl@unimelb.edu.au (B.J.L.); jcottrell@unimelb.edu.au (J.J.C.)

**Keywords:** lactation, trimethylglycine, rumination, heat stress, automatic milking system

## Abstract

**Simple Summary:**

Heat events during summer can result in dramatic reductions in milk production in grazing dairy cows as they attempt to reduce their accumulated heat load. Therefore, there is interest in dietary manipulations that can decrease heat production or increase heat dissipation. One of the actions of sugar beet-derived betaine is to act as an osmolyte and reduce intracellular ion pumping and heat production. Therefore, this study was conducted to investigate the effects of dietary betaine supplementation on milk and milk component production in grazing dairy cows during hot periods in summer.

**Abstract:**

Betaine is an organic osmolyte sourced from sugar beet that accumulates in plant cells undergoing osmotic stress. Since the accumulation of betaine lowers the energy requirements of animals and, therefore, metabolic heat production, the aim of this experiment was to investigate if betaine supplementation improved milk yield in grazing dairy cows in summer. One hundred and eighteen Friesian × Holstein cows were paired on days in milk and, within each pair, randomly allocated to a containing treatment of either 0 or 2 g/kg natural betaine in their concentrate ration for approximately 3 weeks during February/March 2015 (summer in Australia). The mean maximum February temperature was 30 °C. Cows were allocated approximately 14 kg dry matter pasture and 7.5 kg of concentrate pellets (fed in the milking shed) per cow per day and were milked through an automatic milking system three times per day. Betaine supplementation increased average daily milk yield by over 6% (22.0 vs. 23.4 kg/day, *p* < 0.001) with the response increasing as the study progressed as indicated by the interaction (*p* < 0.001) between betaine and day. Milk fat % (*p* = 0.87), milk protein % (*p* = 0.90), and milk somatic cell count (*p* = 0.81) were unchanged by dietary betaine. However, betaine supplementation increased milk protein yield (677 vs. 719 g/day, *p* < 0.001) and fat yield (874 vs. 922 g/day, *p* < 0.001) with responses again being more pronounced as the study progressed. In conclusion, dietary betaine supplementation increased milk and component yield during summer in grazing dairy cows.

## 1. Introduction

Betaine (trimethylglycine) has many activities that may reduce the effect of heat stress (HS) in lactating dairy cows and improve milk production. For example, betaine (BET) is an osmolyte [1], a methyl donor [2], acts as a molecular chaperone [3], can decrease the susceptibility of microbes to stress [4] and, under some circumstances, has antimicrobial activity [5].

Betaine is transported across cell membranes utilizing a sodium-coupled transporter of the betaine/choline/carnitine transporter family involved in the response to hyperosmotic stress [6]. Betaine has a net neutral charge, but also has polar regions, which allows BET to hold intracellular water molecules against a concentration gradient. The ability of BET to act as an osmolyte decreases the need for ion pumping and so can decrease basal heat production and maintenance requirements, at least in pigs [7,8]. A decrease in basal heat production suggests that dietary BET may be a useful strategy to combat HS.

Dietary BET increases milk yield in lactating dairy cows under thermoneutral conditions [9,10,11,12]. BET has been shown to partially mitigate against HS in sheep [13], chickens [14,15], and beef cattle [16]. However, the effects on lactating dairy cows during HS are equivocal [11,17], perhaps because of the complex dose-dependent responses to BET in different organs of the body [18].

Previous studies with dietary BET in dairy cows during HS were conducted in cattle fed a total mixed ration, and conducted indoors in conventional tie-stall systems or controlled environmental rooms. In many parts of the world, including Australia, dairy cows are predominantly grazing outdoors and exposed to a wide variation in temperatures which may include HS conditions during summer. Grazing cattle may be able to alter eating, activity, and other behaviors to reduce the impact of HS and effects of dietary BET under these conditions are unknown. We hypothesize that dietary BET at approximately 15 g per day will reduce the effect of hot summer conditions on milk and milk component production in dairy cattle grazing summer pasture.

## 2. Materials and Methods

The protocol used in this experiment conformed to all Animal Experimentation Ethics Committee regulations concerning the health and care of experimental animals and was approved by The University of Melbourne Veterinary and Agricultural Sciences Animal Ethics committee (Protocol number 1413431.1).

The study was conducted at the dairy farm located at The University of Melbourne Dookie campus (36°38′ S, 145°71′ E) for 22 days during February 2015 (summer in Australia). The region has a Mediterranean climate with annual average rainfall of 540 mm (1990–2015). The farm grazing area consists of 41 ha predominately made up of perennial ryegrass (*Lolium perenne*) and white clover (*Trifolium repens*), which is irrigated from October to April as required. At the time of the study, the herd consisted of approximately 145 Holstein × Friesian cows with about 80% calving in spring (mid-August to September) and the remainder in autumn (mid-March to mid-May). An automatic milking system (Lely Astronaut; Lely, Maassluis, The Netherlands) was used with the cows voluntarily moving into the dairy for milking. Before entering the lactating herd, each cow was fitted with a neck collar containing an identification transponder collar using the pictorial guide provided by the manufacturer (Lely). This transponder also housed the cow activity and rumination monitors (Qwes-HR, Lely). The cow’s diet consisted of concentrates (commercial cereal grain-based pellets), pasture, and silage and/or hay fed when grazing and concentrates were insufficient pasture to meet the herd’s nutritional requirements. Concentrates were fed during milking in the robot (Lely Astronaut) and cows were allowed to finish their individually allocated ration in a feeding station (Lely Cosmix) after milking. The amount of concentrates fed was calculated for each individual cow based on milk yield, stage of lactation, and pasture availability. Silage was fed predominately on the feedpad. The pasture was divided into three grazing zones, and cows were offered three breaks of pasture each day at equally spaced intervals. The average feed intake for the entire herd for the period around the study is shown in Table 1.

**Figure 1:**
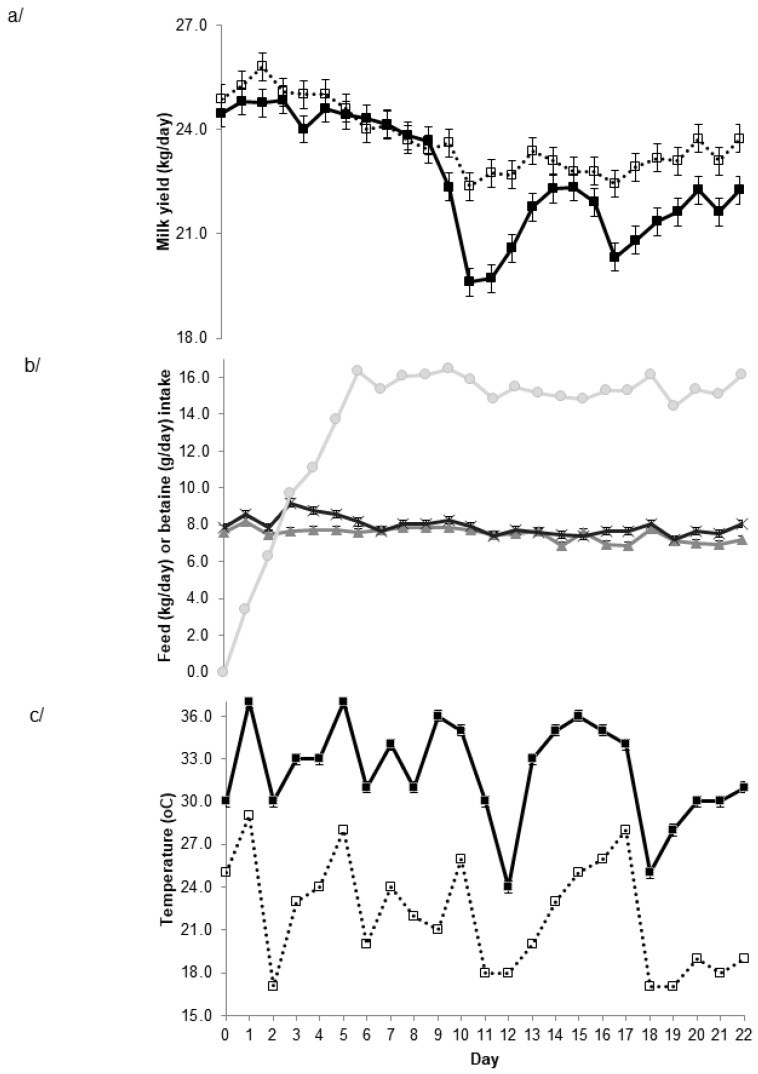
Relationship between day of study and **a**/ milk yield in control (■) and betaine supplemented cows (□), **b**/ concentrate intake (as fed) in control cows (▲) and concentrate (Ӿ) and betaine intake (●) in betaine supplemented cows and **c**/ minimum (□) and maxima (■) temperature recorded at the Dookie College weather station. Values are mean ± s.e.d. for time × treatment for **a**/ and **b**/. For concentrate intake the s.e.d. cannot be seen because they are less than the size of the symbol.

One hundred and eighteen of the spring-calving cows (initial weight 617.0 ± 6.0 kg, mean ± standard error of the mean) from the herd were blocked into 58 pairs based on days in milk and parity and, within each pair, randomly allocated to a concentrate diet containing either 0 or 2 g/kg natural betaine (Feedworks, Lancefield, Australia) for 22 days, with the cows on the BET diet gradually introduced to BET over the first 5 days. The diets were commercially manufactured from identical ingredients (Optimilk, Lacta Max, Rivalea, Corowa, Australia) and to the same specifications (12.5 MJ metabolizable energy (ME)/kg, 16% crude protein (CP)) with the exception of BET, which was added at the expense of cereal grain. The experimental cows were grazed with the rest of the herd as a single herd. The mean maximum and minimum temperatures during the study were 31.8 and 21.7 °C, respectively (Figure 1c). Voluntary milking frequency, milk yield, fat and protein percentage, somatic cell count, and liveweight were recorded automatically using proprietary techniques via the automatic milking system (Lely). Data from the activity and rumination monitors were also downloaded at each milking.

Statistical analysis for the main effects of BET and day, and their interaction on milk yield and components and rumination behavior, was conducted by analysis of variance using residual maximum likelihood (Genstat Version 18, VSN International, Hemel Hemstead, UK), suitable for repeated measures. Block, animal number, and covariate values were used as random factors. Covariates for milk yield and component yield and other variables presented in Table 2 were determined over the three days preceding the commencement of BET feeding. Data analyzed was obtained between days 7 and 23, after which time peak dietary BET was achieved. Data are presented in tabular format to demonstrate the main effects of BET, and in graphical format to demonstrate the main and interactive effects of BET and the day of study. Since the cows were grazed as a single herd dietary treatment, effects on pasture and silage intake could not be determined. However, individual concentrate intake was measured. Data are expressed as means and standard error of the difference (s.e.d.).

## 3. Results

The main effect of dietary BET on milk and milk component yield are presented in Table 2. Dietary BET increased milk (+1.4 kg/day), milk protein (+42 g/day), and milk fat (+48 g/day) yields (Table 2) compared to the control diet. There was no change in milk fat %, milk protein %, or somatic cell count in cows fed supplemental BET. There was also a small increase in voluntary milking frequency in response to supplemental BET (Table 2). Concentrate intake and time spent ruminating were higher in cows receiving supplemental BET.

However, there were significant BET × time interactions for most parameters. For example, there was a day × BET interaction (*p* < 0.001) such that while there was no substantial effect of BET for the first week of the study, there was a substantial response beyond day 10 (Figure 1a). This delay in the initial response may be related to the gradual increase in dietary BET intake over the first week of the study (Figure 1b). Interestingly, the milk yield response beyond day 10 appeared to sometimes occur after a day with a high night time (minimum) temperature (Figure 1c). Milk yield in the control cows decreased at these and other times, whereas it was maintained in the BET-supplemented cows. Qualitatively, similar interactions and responses were observed for milk fat and protein yield (data not shown). There was also an interaction between day and BET (*p* < 0.001) for concentrate intake, although the association with temperature was not as apparent (Figure 1b).

## 4. Discussion

The major finding from this study was that dietary BET supplementation at 15 g/day increased milk and component yield in lactating dairy cows’ grazing pasture during summer, and that the response seemed to manifest as a maintenance of production when the nighttime temperature did not fall to allow dissipation of accumulated heat. At these times, there was a substantial decrease in milk and component production in the control cows. Betaine-supplemented cows also ate more concentrates and spent slightly longer each day ruminating. Elevated temperatures or thermal heat index (THI) during summer are associated with reduced milk production with a lag of between 1 and 3 days [20], and milk production will return to pre-event levels within 5 to 7 days [18]. In the present study, there also appeared to be a similar lag and return to normal production in the control cows, whereas the BET-supplemented cows maintained milk production. Of course, not all of the reduction in milk yield in the control cows may be due to elevated nighttime temperatures, with daytime temperature, humidity, and pasture quality also likely to impact. Regardless of the cause, these effects were not as apparent in those cows supplemented with BET.

A number of researchers have found that up to 150g/day dietary BET increases milk yield in a linear manner under thermoneutral conditions [9,10,11]. However, the effects of dietary BET on lactating dairy cows during summer, or under hot conditions, are more equivocal [11,17], perhaps because of the multifaceted dose-dependent responses to BET [18]. For example, milk responses to dietary BET during HS were maximized at 15 g betaine per day and, indeed, disappeared at doses above this [17]. Consistent with these findings, Hall et al. [11] found that 35 and 70 g betaine per day increased milk yield under thermoneutral conditions, but not during HS. Curvilinear dose responses to dietary betaine have also been observed in sheep and beef cattle during HS being optimized at 2 and 15 g betaine per day, respectively [13,16]. The reason for the moderation in response to high doses of BET during HS may be that the stimulation of hepatic metabolism and resultant increase in heat production the liver may offset the reduction in heat production due to the osmoprotective effects of BET in the rest of the body [18].

In this study, there was an increase in concentrate intake during BET supplementation as well as an increase in time spent ruminating. A greater amount of time spent ruminating in the BET-supplemented cows would suggest that the higher concentrate intake was not compromising rumen function, and that the cows were receiving adequate long fiber [21]. All cows received silage and grazed pasture as a single herd so effects of BET on total feed intake cannot be determined. Nevertheless, consumption of rapidly fermented grain concentrates increases the risk of HS [22,23] and, therefore, it appears that the osmoprotective effects of dietary BET must have offset any increased heat production through increased concentrate intake.

Another possible site of action of dietary BET is within the rumen. Dietary BET increased volatile fatty acid production [9] so it is possible that dietary BET can enhance rumen fermentation. The osmoprotective effects of BET are observed in microbial populations and BET has been shown to promote favorable bacterial growth under osmotic stress conditions [24,25], including fluctuations in pH [26], and this may extend to HS conditions since rumen temperature increases during HS [20,22,23]. While rumen microbial utilization and degradation of BET would limit availability to rumen epithelial or other nondigestive tract cell types, dietary BET is present in the duodenum indicating that some BET escapes the rumen [27]. Also, some of the BET metabolized in the rumen is converted into acetate, which may contribute to milk fat synthesis [10]. While we do not have any measure of enhanced rumen activity in BET-supplemented cows, the greater amount of time spent ruminating indicates that rumen function may be improved.

This study was conducted in a grazing production system that utilized an automated milking system which utilized three grazing zones where cows were offered three breaks of pasture each day. Consequently, cows were milked approximately three times per day, although the BET-supplemented cows had slightly more milkings per day, most likely because of a greater demand to eat concentrates or to be milked. Whether the decreased heat production in BET-supplemented cows drove increased concentrate intake, milk production, and milking frequency is still to be determined.

## 5. Conclusions

These data clearly show that 15 g/d of dietary BET can increase milk and component yield in grazing dairy cattle during hot periods in summer. The response to dietary BET appears to be most pronounced at times when milk yield decreased in the control cows, perhaps because of environmental conditions impacting the animal or pasture. Cows supplemented with dietary BET had higher concentrate intakes and spent slightly more time ruminating than their control counterparts.

## Figures and Tables

**Table 1:** Concentrate, silage, and estimated pasture dry matter (DM) intake for the entire herd for the six months from November 2014.

Month	Concentrate Intake ^1^ (kg DM/cow.day)	Silage Intake ^2^ (kg DM/cow.day)	Pasture Intake ^3^ (kg DM/cow.day)	Temperature ^4^ (°C)
November 2014	5.7	1.3	12.5	19 (5–34)
December 2014	6.4	4.3	9.5	21 (6–34)
January 2015	6.7	3.7	9.2	22 (8–37)
February 2015	6.7	4.1	7.5	24 (15–37)
March 2015	6.1	6.0	6.0	17 (5–30)
April 2015	8.1	8.9	4.2	15 (5–29)

^1^ Providing 12.5 MJ ME/kg DM, 16% CP. ^2^ Providing an average of 8.5 ME MJ/kg DM, 14% CP. ^3^ Back-calculated from herd concentrate, silage intake, and milk production using the method of [19]. ^4^ Average (minimum − maximum) temperature. The study was conducted in February 2015.

**Table 2:** Main effect of dietary betaine (approximately 15 g/day) on milk and milk components. Data are means and between day 7 and day 23 after peak betaine (BET) intake was achieved.

Parameter	Control (*n* = 58)	Betaine (*n* = 58)	s.e.d.	*p*-Value
Milk yield (kg/day)	22.0	23.4	0.28	<0.001
Milk protein (%)	3.14	3.13	0.004	0.90
Milk protein yield (g/day)	677	719	3.5	<0.001
Milk fat (%)	4.10	4.10	0.008	0.87
Milk fat yield (g/day)	874	922	4.2	<0.001
Somatic cell count	59.1	57.6	7.98	0.81
Milking frequency (milking/day)	3.13	3.21	0.006	<0.001
Concentrate intake (kg DM/day)	6.49	6.75	0.045	<0.001
Rumination (min/day)	453	465	2.6	<0.001

s.e.d.: standard error of the difference.
